# Supplementary material for: Surgical Treatment of Corneal Shield Ulcer in Vernal Keratoconjunctivitis: A Systematic Review
Source: J Pers Med. 2023 Jul 2;13(7):1092. doi: 10.3390/jpm13071092 (PMC10381665; doi:10.3390/jpm13071092)
Supplement: Supplementary file 1 [file jpm-13-01092-s001.zip › jpm-2458802-supplementary.pdf]

Supplementary file 1. Details of literature search across databases.

PubMed:

History and Search Details

Download
Delete

Search	Actions	Details	Query	Results	Time
#1			Search: **"Vernal Keratoconjunctivitis"[All Fields] AND "shield ulcer"[All Fields]** Sort by: **Most Recent** "Vernal Keratoconjunctivitis"[All Fields] AND "shield ulcer"[All Fields]	38	13:47:30

Showing 1 to 1 of 1 entries

Embase:

Search History
(7searches found)

Contract	# ▲	Searches	Results	Type	Actions	Annotations
	1	"Vernal Keratoconjunctivitis".mp. [mp=title, abstract, heading word, drug trade name, original title, device manufacturer, drug manufacturer, device trade name, keyword heading word, floating subheading word, candidate term word]	1140	Advanced	Display Results More	
	2	vernal conjunctivitis/	1378	Advanced	Display Results More	
	3	"shield ulcer".mp. [mp=title, abstract, heading word, drug trade name, original title, device manufacturer, drug manufacturer, device trade name, keyword heading word, floating subheading word, candidate term word]	65	Advanced	Display Results More	
	4	cornea ulcer/	8331	Advanced	Display Results More	
	5	1 or 2	1657	Advanced	Display Results More	
	6	3 or 4	8367	Advanced	Display Results	

				More	
7	5 and 6	181	Advanced	Display Results More	

Cochrane Central:

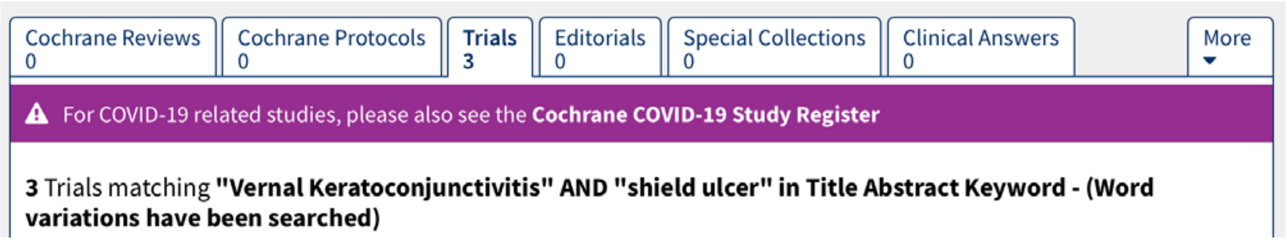

Web of Science mfl.

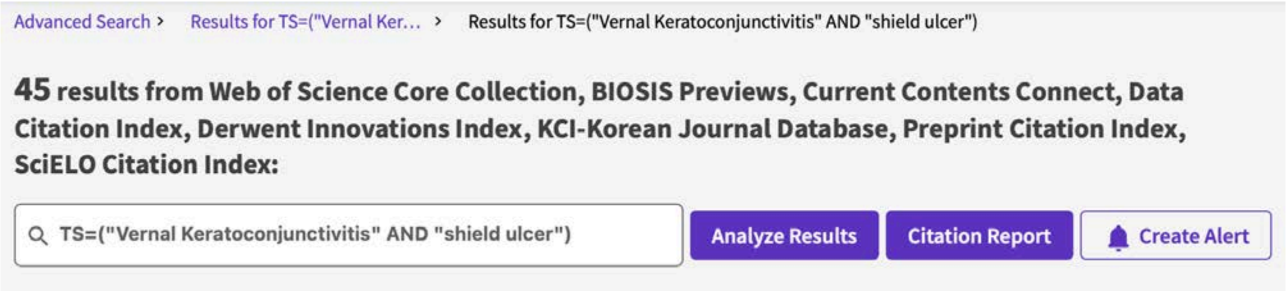

ClinicalTrials.Gov

NIH U.S. National Library of Medicine

ClinicalTrials.gov

Find Studies ▾ About Studies ▾ Submit Studies ▾ Resources ▾ About Site ▾

PRS Login

Home > Search Results

Saved Studies (8)

Modify Search Start Over

1 Study found for: **shield ulcer | Vernal Keratoconjunctivitis**
